# Applying Engagement Frameworks to Case Scenarios: A Retrospective Analysis

**DOI:** 10.1093/geroni/igaa057.2858

**Published:** 2020-12-16

**Authors:** Erin McGaffigan

**Affiliations:** Gerontology Institute, North Reading, United States

## Abstract

The LeadingAge LTSS Center @UMass Boston has worked with the Patient-Centered Outcomes Research Institute (PCORI), service recipients, researchers, and providers since 2017 to understand the obstacles and creative solutions for meaningful and effective engagement in research. The LTSS Center’s work has been informed by multiple engagement frameworks, including Dr. McGaffigan’s 2011 PAE Attention Framework, the result of a multi-state, multi-site research study examining the factors to user engagement in Cash & Counseling programs. In this presentation, we will apply the PAE Attention Framework (2011), the PCORI Engagement Rubric (2014), and the NHS INVOLVE Framework (2015) to three research study case scenarios to understand the strategies used, outcomes realized, and factors influencing engagement success. Lessons learned from each and their application to future research will be discussed, including ethical considerations. Part of a symposium sponsored by the Patient/Person Engagement in Research Interest Group.

